# Loss of serum response factor in mature neurons in the dentate gyrus alters the morphology of dendritic spines and hippocampus-dependent behavioral tasks

**DOI:** 10.1007/s00429-019-01925-6

**Published:** 2019-08-02

**Authors:** Karolina Nader, Anna Krysiak, Anna Beroun, Martyna Pekala, Magda Szymanska, Bozena Kuzniewska, Kasia Radwanska, Leszek Kaczmarek, Katarzyna Kalita

**Affiliations:** 1 Laboratory of Neurobiology, Nencki Institute of Experimental Biology, Polish Academy of Sciences, 3 Pasteur Street, 02-093 Warsaw, Poland; 2 Laboratory of Molecular Basis of Behavior, Nencki Institute of Experimental Biology, Polish Academy of Sciences, 3 Pasteur Street, 02-093 Warsaw, Poland

**Keywords:** Serum response factor, Cofilin 1, Dentate gyrus, Structural plasticity, Rodent-specific behaviors

## Abstract

Serum response factor (SRF) is a major transcription factor that regulates the expression of several plasticity-associated genes in the brain. Although the developmental expression of SRF in excitatory neurons is crucial for establishing proper hippocampal circuitry, no substantial evidence of its role in unstimulated mature neurons has been provided. The present study used time-controlled, conditional SRF knockout mice and found that the lack of SRF in adult neurons led to decreased actin levels and inactivation of the actin-severing protein cofilin 1 through its increase in phosphorylation at Ser3. The augmentation of cofilin 1 phosphorylation correlated with an alteration of dendritic spine morphology in the dentate gyrus, which was reflected by an increase in the number of spines that clustered into the long-spine category. The changes in spine morphology coincided with a lower amplitude and frequency of miniature excitatory postsynaptic currents. Moreover, SRF knockout animals were hyperactive and exhibited impairments in hippocampus-dependent behaviors, such as digging, marble burying, and nesting. Altogether, our data indicate that the adult deletion of neuronal SRF leads to alterations of spine morphology and function and hippocampus-dependent behaviors. Thus, SRF deletion in adult neurons recapitulates some aspects of morphological, electrophysiological, and behavioral changes that are observed in such psychiatric disorders as schizophrenia and autism spectrum disorders.

## Introduction

Serum response factor (SRF) is a transcription factor that is highly expressed in granular neurons in the dentate gyrus (DG) of the hippocampus (Etkin et al. 2006; Ramanan et al. 2005). It regulates the expression of many cytoskeletal genes and genes that are activated by neuronal stimulation (Benito et al. 2011; Etkin et al. 2006; Kalita et al. 2006; Kuzniewska et al. 2016; Losing et al. 2017; Miano et al. 2007; Parkitna et al. 2010; Ramanan et al. 2005; Treisman 1995). During brain development, SRF-regulated genes control the neuronal cell migration, neurite outgrowth and organization of the DG and the formation of mossy fiber circuitry (Alberti et al. 2005; Etkin et al. 2006; Knoll et al. 2006; Lu and Ramanan 2011; Scandaglia et al. 2015; Stritt and Knoll 2010). The early postnatal deletion of *Srf* resulted in impairments in long-term potentiation and long-term depression and alterations of the formation of immediate memory of a novel context (Etkin et al. 2006; Ramanan et al. 2005). In contrast to early SRF knockout (KO), time-specific SRF deletion in adult excitatory neurons does not influence gross morphology of the hippocampus (Kuzniewska et al. 2016; Losing et al. 2017). Moreover, SRF deletion in the mature brain caused a very limited alteration of basal gene expression, restricted mostly to genes that encode the actin cytoskeleton (Kuzniewska et al. 2016; Losing et al. 2017; Parkitna et al. 2010). Nevertheless, these mice exhibit hyperactivity, a decrease in anxiety-like behavior, and epilepsy (Kuzniewska et al. 2016; Losing et al. 2017; Zimprich et al. 2017).

The DG is part of the hippocampal formation and mostly composed of granule cells (GCs) (Kempermann et al. 2004; Radic et al. 2017). The DG receives excitatory inputs from the entorhinal cortex, and GCs send excitatory outputs via mossy fibers to pyramidal cells in the Cornu Ammonis CA3 (Amaral et al. 2007). Alterations of hippocampal DG circuity have recently been implicated in the pathophysiological mechanisms of psychiatric disorders (Hagihara et al. 2013; Yu et al. 2014).

To investigate whether SRF controls the morphology and function of mature GCs, we employed a model of SRF depletion that was restricted to excitatory neurons (Kuzniewska et al. 2016). In the present study, we found that the ablation of SRF in adult GCs decreased β-actin levels and led to cofilin 1 inactivation through an increase in the phosphorylation at regulatory Ser3. The decrease in actin expression coincided with abnormal spine morphology and impairments in basal excitatory synaptic transmission in DG neurons. Moreover, the lack of SRF increased animal activity and impaired rodent-typical behaviors, such as digging, marble burying, and nesting. Altogether, our data indicate that SRF expressed in adult excitatory neurons plays a crucial role in maintaining actin expression and controlling the morphological and electrophysiological properties of granular neurons and innate animal behaviors.

## Materials and methods

### Animals

Conditional Srf KO mice (mutant mice; *Srf*^f/f*CaMKCreERT2*^) were generated as previously described (Kuzniewska et al. 2016). As a control, CreERT2-negative littermates were used (*Srf*^f/f^; control mice; wild type [WT]). The mice were bred on a C57BL/6J genetic background. Adult male and female *Srf*^f/f^ and *Srf*^f/f*CaMKCreERT2*^ mice were intraperitoneally injected with 1 mg tamoxifen (TAM; cat. #T5648, Sigma) twice daily for 10 days, resulting in the translocation of Cre-recombinase to the nucleus. Experiments were performed at least 6 weeks after the TAM injection. The animals were bred in the Animal House of the Nencki Institute of Experimental Biology. The mice were housed in individual cages with a 12 h/12 h light/dark cycle, constant temperature (23–25 °C), and food and water available ad libitum. Both male and female mice were used for the experiments, except when marked otherwise. All of the experiments were performed by experimenters who were blind to mouse genotype. All of the work was conducted in accordance with the European Community Council Directive (86/609/EEC) and Animal Protection Act of Poland (Directive 2010/63/EU). The procedures were approved by the 1st Local Ethics Committee in Warsaw, Poland (permission no. 389/2012, 678/2015, and 144/2016).

### RNA preparation and quantitative real-time polymerase chain reaction

Total RNA was isolated from the mouse hippocampus using the RNeasy Mini Kit (cat. #74104; Qiagen) as described by the manufacturer. DNA contamination was removed by digestion with DNase I (cat. #1023460, Qiagen). The RNA concentration was calculated from absorbance at 260 nm, and the 260/280 nm absorbance ratio determined the purity of RNA. RNA was reverse transcribed with SuperScript III or IV Reverse Transcriptase (cat. #18080-044, #18090050, Invitrogen) according to the manufacturer’s instructions. Quantitative real-time polymerase chain reaction (PCR) was performed using Fast TaqMan Master Mix (cat. #44456, Applied Biosystems) with an Applied Biosystems 7900HT Fast Real-Time PCR System using TaqMan probes (ThermoFisher). Fold changes in expression were determined using the ∆∆C_T_ relative quantification method. The values were normalized to relative amounts of GAPDH.

### Immunohistochemistry

The animals were perfused with 4% paraformaldehyde (PFA). Brains were then fixed in 4% PFA overnight at 4 °C and cut into 40-μm coronal slices. For SRF staining, brain slices were stained with anti-SRF antibody (1:500; cat. #sc-13029; Santa Cruz Biotechnology) overnight at 4 °C followed by avidin/biotin complex (cat. #PK-6100, Vector) and visualized using SIGMAFAST™ DAB (cat. #D0426, Sigma). For Nissl staining, the brain sections were stained with 0.1% cresyl violet solution and 3% acetic acid for 5 min, washed, dehydrated, cleared in xylene, and coverslipped.

### Western blot

Twenty micrograms of protein extracts were run on polyacrylamide gels (cat. #4569033, BioRad). The standard procedure of Western blot was performed using anti-Ser 3-phospho cofilin 1 and cofilin 1 (cat. #3313, #5175, Cell Signaling), and β-actin (cat. #A1978, Sigma) antibodies. To ensure equal total proteins level, blots were reprobed with tubulin (cat. #T9026, Sigma) antibodies. For detection, the chemiluminescent method was used. To quantify individual bands, a scan of photographic films was analyzed by densitometry with GeneTools Software (Syngene).

### Electrophysiology

Whole-cell patch-clamp recordings were carried out in voltage-clamp mode. Mice (male and female) were anesthetized with isoflurane and decapitated. Brain slices (coronal, 250 μm thick) prepared using Leica VT 1200S vibratome in ice-cold NMDG solution (135 mM NMDG, 1.2 mM KH_2_PO_4_, 1 mM KCl, 1.5 mM MgCl_2_·6H_2_O, 0.5 mM CaCl_2_·2H_2_O, 20 mM choline bicarbonate, 10 mM d-glucose, saturated with carbogen—95% O_2_, 5% CO_2_) were then transferred to the artificial cerebrospinal fluid (ACSF) solution (119 mM NaCl, 2.5 mM KCl, 1.3 mM MgCl_2_·6H_2_O, 1 mM NaH_2_PO_4_, 26 mM NaHCO_3_, 20 mM d-glucose, 2.5 mM CaCl_2_·2H_2_O, saturated with carbogen), incubated for 12 min at 31 °C and then at least 1 h at room temperature. During recordings, slices were held in a recording chamber perfused with carbogenated ACSF solution supplemented with 100 μM picrotoxin and 0.5 μM tetrodotoxin and heated up to 31 °C. Granule cells of the upper blade of dentate gyrus were identified visually. Borosilicate glass capillaries (4–7 MΩ resistance) used for patch-clamp recordings were filled with Cs-based internal solution (130 mM Cs-gluconate, 20 mM HEPES, 0.4 mM EGTA, 3 mM TEA-Cl, 4 mM Na_2_ATP, 0.3 mM NaGTP, 4 mM QX-314Cl; osmolarity: 285–290 mOsm, pH = 7.0–7.1). Data were acquired using custom algorithms in Igor Pro (Wavemetrics) with an NPI amplifier and digitized at 10 kHz with an ITC-18 InstruTECH/HEKA. Recorded currents were filtered at 2 kHz. Series and input resistances were monitored throughout the experiment. To measure miniature excitatory postsynaptic currents (mEPSCs) 10- to 20-min-long voltage-clamp recordings were collected. Miniature events were analyzed using the MiniAnalysis software (Synaptosoft). mEPSCs amplitude detection threshold was set to 7 pA. All mini events automatically detected by the software were verified visually by the experimenter.

### Analysis of dendritic spine morphology

Serum response factor KO and WT mice (male and female *n* = 8) were anesthetized and transcardially perfused with phosphate-buffered saline (PBS) and with 1.5% paraformaldehyde (PFA) at room temperature. The brains were placed in 1.5% PFA for 20 min for postfix and then transferred to ice-cold PBS for at least 20 min. Next, these brains were cut on a vibratome into 130-µm-thick slices and placed in PBS at room temperature for 1 h. Gene gun technique was used to label sections with tungsten particles (Bio-Rad, Hercules, CA, USA) coated with lipophilic dye DiI (1,1ʹ-dioctadecyl-3,3,3ʹ,3ʹ-tetramethyl indocarbocyanine perchlorate; d-3911, cat. #D282, ThermoFisher). The slices were incubated in PBS at the room temperature for about 3 h and next in 1.5% PFA in 4 °C overnight, which enables diffusion of the dye into neuronal processes and allows to visualize dendritic spines. Z-stacks of confocal images of the seven dendrites per animal from the middle molecular layer of the upper blade of dorsal DG were acquired with 561-nm laser line with Zeiss LSM780 confocal system. Neurons of immature morphology (very sparse dendritic spines) were excluded from the analysis. The semiautomatic SpineMagick! software was used to measure and analyze dendritic spines by obtaining the virtual skeletons (Ruszczycki et al. 2012). We used a scale-free parameter (the length/width ratio) which reflect the spine shape (Michaluk et al. 2011). Length/width ratio was calculated and plotted using a logarithmic scale. The density of spines was calculated as a number of spines per 1 μm of dendrite length. Spines’ shapes were divided into clusters and then sorted into two groups: “long” and “mushroom and stubby” spines using custom scripts (Jasinska et al. 2016).

### Behavioral tests

*Open field* The apparatus had a wooden floor (50 cm × 50 cm) surrounded by walls (34 cm hight). Animals’ behavior was monitored by a video camera placed above the center of the apparatus. Adult females (WT *n* = 12, SRF KO *n* = 12, about 6 months old or older) were put individually in one corner of the open field facing the wall and were allowed to explore freely for 15 min. The floor of the apparatus was cleaned with 5% ethanol after each session. Data were analyzed using EthoVision 3.1 System (Noldus Information Technology), total distance traveled was counted.

*Species*-*typical behaviors* All species-typical behaviors tests were performed on the same group of animals (WT *n* = 10, SRF KO *n* = 7; adult female and male, about 6 months old or older), starting from overnight nesting test and then marble burying and digging.

*Nesting* Mice were housed individually in their home cages with standard bedding for at least 1 week. After that time paper towel (divided into six pieces 9 cm × 9 cm each) was placed in the middle of each cage and left overnight to assess the nest building ability of the mice. The following scoring system was used: (1) paper towel was mostly untouched (> 90%) and was left in the middle of the cage; (2) flat pad-shaped nest, mostly moved to a corner; (3) more complex nest with biting the towel but not gathered in one place; (4) nest with shredded paper to form a cup; (5) perfect nest, paper towel torn up to form a crater, where walls are higher than mouse body height. Nest building is spontaneous home cage behavior of mice. It indicates healthy functioning and well-being of an animal (Jirkof 2014; Moretti et al. 2005). It is also interpreted by some authors as social behavior (Crawley 2012).

*Marble burying* Clean cage was filled 5 cm deep with bedding, tamped down to make even and flat surface. The same 12 glass marbles (15 mm in diameter) were placed in a regular pattern in two rows on bedding surface. Mice were placed individually into the experimental cage for 30 min. After that time, mice were taken to their home cages, and the number of marbles buried to at least two-thirds of their depth with bedding was counted. The same cage was re-used for all tested animals.

*Digging* Clean cage was prepared with the flat surface of wooden, 5-cm-deep bedding. Mice were placed individually in the experimental cage. The duration of the test was 3 min and latency to start digging, a number of digging bouts and total duration of digging were recorded and analyzed. Coordinated movements of force and/or hindlimbs which displace the wooden bedding defined the digging. The new bedding was used for all tested animals. Marble burying measures spontaneous digging behavior, which is typical for mice and is dependent on hippocampal function (Deacon 2006; Deacon and Rawlins 2005).

### Statistical analyses

To compare the distributions, Shapiro–Wilk normality test was performed. To test the differences between two groups, unpaired *t* test or Mann–Whitney test (nonparametric) was used. When required repeated-measures ANOVA was performed, followed by Bonferroni’s multiple comparisons post hoc test. To compare the cumulative distributions of mEPSCs amplitudes and frequencies, the Kolmogorov–Smirnov test was used. The number of animals and neurons which were used for analysis is provided in figures legend. Data on the graphs are expressed as cumulative probability or means ± standard errors of the means (SEM). The difference between the experimental groups was considered as significant when *p* < 0.05. Results were analyzed in GraphPad Prism software.

## Results

### Decrease in β-actin expression in hippocampal neurons in SRF KO animals

**Fig. 1:**
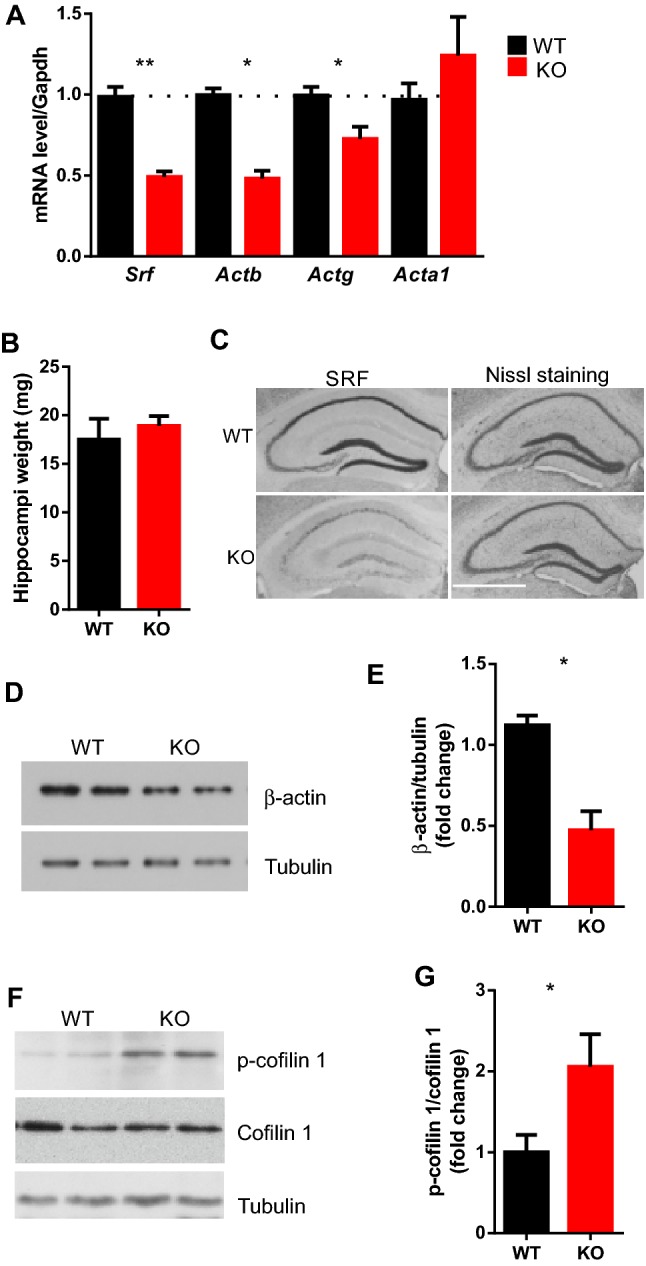
The lack of SRF does not affect the general architecture of the adult hippocampus despite changes of actin cytoskeletal components. **a** Quantitative real-time RT-PCR analysis of *Srf, Actb*, and *Actg* and *Acta1* mRNA levels in the hippocampus in control wild-type littermates (WT) and knockout mice (KO; *n* ≥ 4 for each genotype; Mann–Whitney nonparametric test, SRF, **p = 0.0079; Actb, **p = 0.0079, Actg, *p = 0.0159, Acta1, *p *= 0.5556, ns). **b** Hippocampus weight analysis in control and SRF KO animals (*n* = 6 for each genotype; Mann–Whitney nonparametric test, *p *= 0.3000, ns). No differences in total hippocampus weight were found between genotypes. **c** Nissl staining of brain sections from WT and SRF KO animals. **d** Western blot analysis with β-actin antibody. Each line represents a single animal. **e** Downregulation of β-actin protein in DG extracts from SRF KO animals (*n *≥ 4; Mann–Whitney nonparametric test, *p = 0.0159). **f** Western blot analysis with p-cofilin 1 and cofilin 1 antibody. Each line represents a single animal. **g** Increase in cofilin 1 phosphorylation in DG extracts from SRF KO mice relative to total cofilin 1 level (*n* ≥ 4; Mann–Whitney nonparametric test, *p = 0.0175)

The genome-wide analysis of basal gene expression in animals with the adult deletion of neuronal SRF showed changes in mRNA that were restricted to genes that encode the actin cytoskeleton, such as *Actb* and *Actg1* (Kuzniewska et al. 2016; Losing et al. 2017; Parkitna et al. 2010). To confirm the downregulation of actin expression in the hippocampus in SRF KO animals, we used inducible conditional *Srf* KO mice to ablate *Srf* exclusively in adult, excitatory forebrain neurons (Erdmann et al. 2007; Kuzniewska et al. 2016). *Actb*, *Actg1*, and *Acta1* mRNA levels were analyzed in the hippocampus in mutant and control littermates using quantitative real-time PCR. We found decreases in the levels of mRNA of β-actin and non-muscle γ-actin in the hippocampus in SRF KO animals, but not in smooth muscle α-actin (Fig. 1a).

To assess the consequences of SRF depletion in adult neurons, we focused on the morphology of the hippocampus. Using Nissl staining, we confirmed the lack of gross neuroanatomical defects in SRF KO animals with low SRF expression in the hippocampus (Fig. 1c) (Kuzniewska et al. 2016). We also analyzed the size of the hippocampal formation in adult WT and SRF KO animals. No differences were found in the total weight of the hippocampus (Fig. 1b; Mann–Whitney test, *p* > 0.05).

We performed Western blot assay to confirm actin downregulation in DG neurons. We observed a ~ 50% drop in β-actin protein expression levels in the DG in SRF-deficient neurons (Fig. 1d, e). In non-neuronal cells, the downregulation of β-actin induces cofilin 1 phosphorylation at Ser3, leading to its inhibition (Liu et al. 2007). Cofilin 1 is an actin-binding protein that regulates actin filament dynamics, stimulating the severance of actin filaments. To test whether the low level of β-actin and γ-actin in adult neurons correlates with changes in the actin-binding proteins, we examined the expression of cofilin 1 in the DG. Western blot analysis revealed that cofilin 1 phosphorylation was upregulated in SRF KO DG extracts (Fig. 1f) and coincided with a lower level of β-actin (Fig. 1d).

### Morphology of dendritic spines in granular neurons that lack SRF

**Fig. 2:**
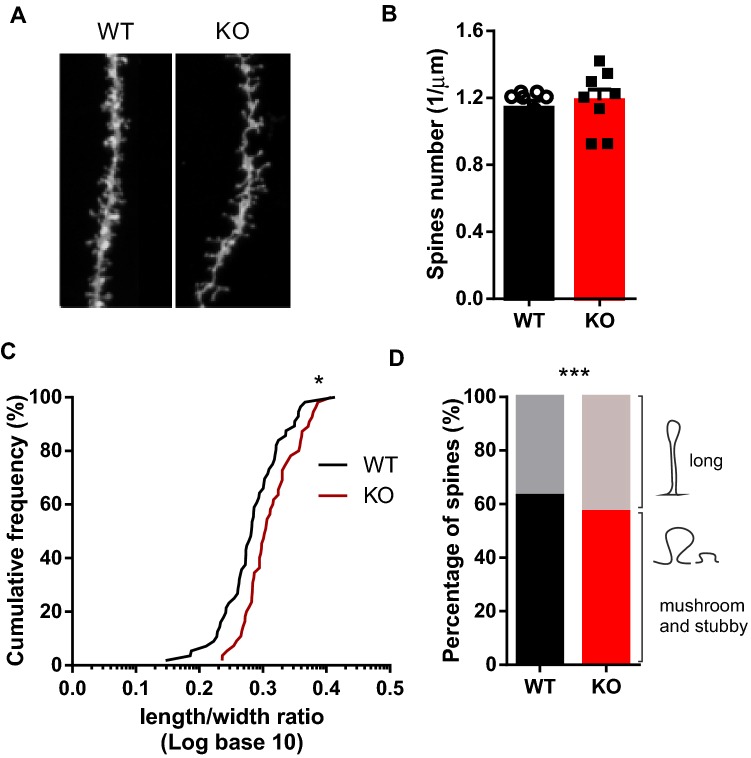
Serum response factor expression in mature excitatory neurons is essential for maintaining the proper structure of dendritic spines. **a** Example photographs of DiI-stained dendrites with dendritic spines of granule neurons from the dentate gyrus in WT and SRF KO animals (*n* = 8 animals, males and females). **b** Dendritic spine density of DG neurons in WT and SRF KO animals (*t* test, *p *= 0.6374, ns). **c** Cumulative histogram of dendritic spine length-to-width ratio (log) in WT and SRF KO DG neurons. SRF KO mice had a higher length-to-width ratio than WT mice (Kolmogorov–Smirnov test, *p = 0.0244). **d** Percentage of protrusions clustered into two categories (long spines vs. mushroom and stubby spines) in the DG (*χ*^2^ = 24, *df* = 29.1, *p* < 0.001). Dendritic spine number and morphology were analyzed using semiautomated SpineMagic! software. Spine clustering was performed using custom scripts

Actin dynamics play a crucial role in neurons, especially in the regulation of dendritic spine morphology (Hotulainen and Hoogenraad 2010; Hotulainen et al. 2009). Therefore, we investigated whether a decrease in β-actin protein expression and increase in cofilin 1 phosphorylation affect dendritic spine morphology. We evaluated the effects of SRF deficiency on the density and morphology of dendritic spines of GCs in the molecular layer of the dorsal DG (Fig. 2a). The analysis of spine density showed no significant changes in the number of spines of DG neurons (upper blade; Fig. 2b; *t* test, *p* > 0.05). To assess whether SRF depletion modified dendritic spine morphology, we measured the length and width of the spines to evaluate spine shape (Michaluk et al. 2011). The cumulative probability graph shows a significant increase in length-to-width ratio in DG neurons from SRF KO animals (Fig. 2c; Kolmogorov–Smirnov test, *p* < 0.001) which testify change towards longer and thinner dendritic spines. To further investigate the way in which SRF depletion affects dendritic spine shape, we clustered spines into two morphological categories: long spines vs. mushroom and stubby spines (Fig. 2d) (Jasinska et al. 2016). Serum response factor KO mice exhibited a significant increase (~ 6 percentage points) in the frequency of long spines in the DG and a significant decrease in the population of mushroom and stubby spines (*χ*^2^ test, *p* < 0.0001) compared with WT animals.

**Fig. 3:**
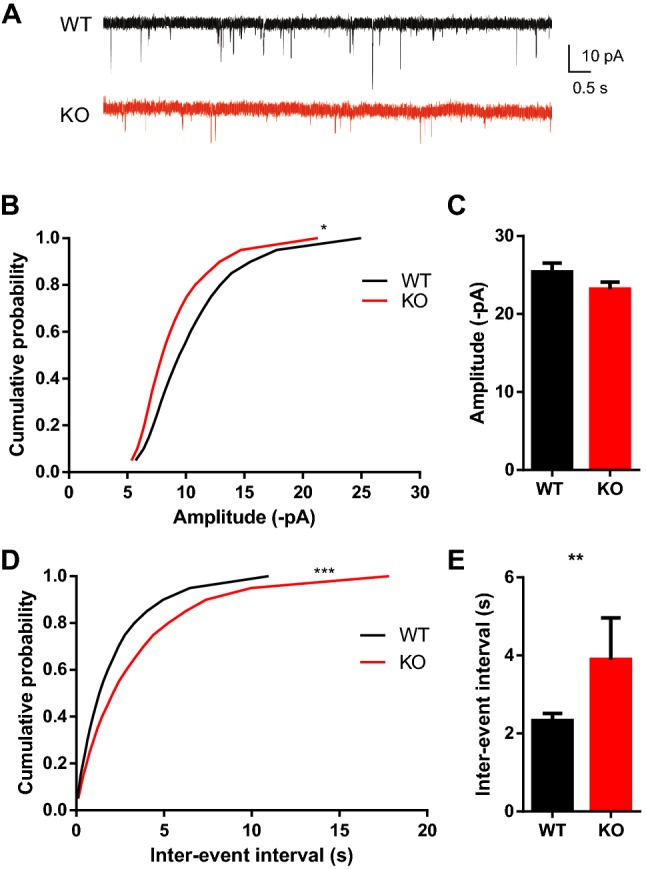
Reduction of the amplitude and frequency of spontaneous mEPSCs in SRF KO granular neurons. **a** Sample traces from WT and SRF KO granular neurons. Cumulative probability plots showing mEPSCs amplitude (**b)** and frequency (**d**) in *n* = 16 WT cells and *n *= 14 SRF KO neurons (Kolmogorov–Smirnov test, *p = 0.01 and ***p < 0.001, respectively). Bar graphs in **c** and **e** represent averaged amplitudes and frequencies of mEPSCs (Mann–Whitney test, *p* = 0.1246, ns and **p = 0.003, respectively)

The morphology of dendritic spines under many conditions is highly correlated with changes in synaptic strength (Engert and Bonhoeffer 1999; Kasai et al. 2003; Yuste and Bonhoeffer 2001). To test whether SRF deletion affects basal glutamatergic transmission in adult neurons, α-amino-3-hydroxy-5-methyl-4-isoxazolepropionic acid (AMPA) receptor-mediated miniature excitatory postsynaptic currents (mEPSCs) were measured (Fig. 3). The comparison of cumulative probability of cellular responses between WT mice and SRF KO mice demonstrated a significant decrease in mEPSC amplitude (Fig. 3b; Kolmogorov–Smirnov test, *p* < 0.001) and frequency (Fig. 3d; Kolmogorov–Smirnov test, *p* = 0.01).

### Serum response factor deletion in mature neurons induces hyperactivity and alterations of species-typical behaviors

**Fig. 4:**
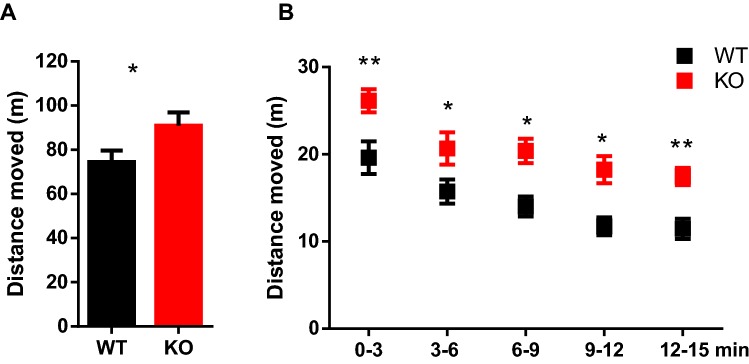
SRF KO mice exhibit increase in spontaneous activity in the open field test. Mice individually explored the open field for 15 min. **a** Distance traveled in 15 min by WT and SRF KO mice *n *= 12 WT, *n *= 12 SRF KO female adult animals (*t* test, *p = 0.0484). **b** Mutant animals were significantly more active in all 3-min time bins in the open field (two-way repeated-measures ANOVA: genotype: *F*_1,11_ = 25.63, *p* = 0.0004, time: *F*_4,44_ = 21.28, *p* < 0.0001, genotype × time: *F*_4,44_ = 0.1032, *p* = 0.9808; followed by Sidak multiple-comparison post hoc test: *p < 0.05, **p < 0.01)

To determine whether changes in the morphology and function of DG neurons in SRF KO animals correlate with behavioral deficits, we compared locomotor activity in WT and SRF KO animals in the open field test. Serum response factor KO animals were significantly more active than WT animals during a 15-min exploration session (Fig. 4a). Differences between genotypes were apparent from the first minute of exploration until the end of the test session (Fig. 4b; repeated-measures analysis of variance [ANOVA], genotype: *F*_1,11_ = 25.63, *p* = 0.0004, time: *F*_4,44_ = 21.28, *p* < 0.0001, genotype × time: *F*_4,44_ = 0.1032, *p* = 0.9808, ns).

**Fig. 5:**
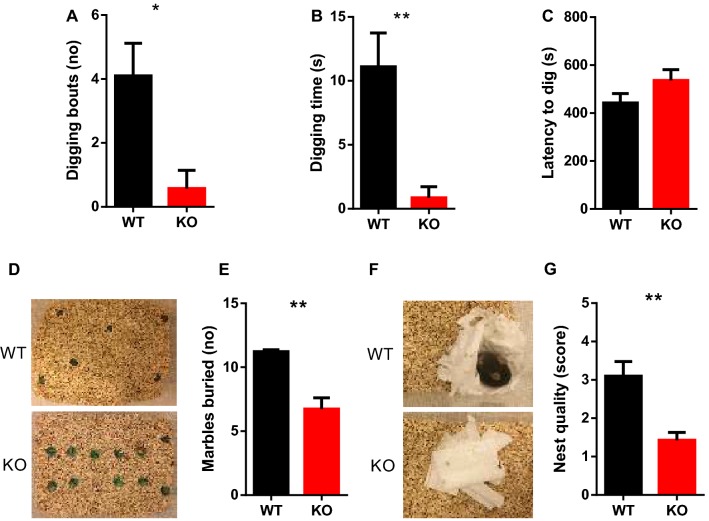
Non-cognitive-dependent behaviors are impaired in SRF KO animals. Species-typical behavioral tasks were analyzed (*n *= 10 WT, *n* = 7 SRF KO). **a** SRF KO mice made fewer digging bouts (Mann–Whitney *t* test, *p = 0.0154). **b** The time spent digging was shorter in SRF KO mice (Mann–Whitney *t* test, **p = 0.0086). **c** Latency to start digging did not differ between genotypes (Mann–Whitney *t* test, *p* = 0.1748, ns). **d** Photographs of cages after 30-min session. **e** The number of marbles that were buried was less in SRF KO mice than in WT controls (Mann–Whitney *t* test, **p = 0.0047). **f** Photographs of cages after nest building test. **g** SRF KO animals built less complex nests than WT animals (Mann–Whitney *t* test, **p = 0.0070)

Next, we evaluated the role of SRF using simple, hippocampus-dependent tasks, including digging, marble burying, and nest building (Deacon 2006; Deacon et al. 2002). In the digging paradigm, SRF KO animals performed fewer digging bouts (Fig. 5a; Mann–Whitney test, *p* < 0.05). Serum response factor KO animals also spent less time digging than control animals (Fig. 5b; Mann–Whitney test, *p* < 0.01). No difference in the latency to start digging was found between KO and WT animals (Fig. 5c; Mann–Whitney test, *p* > 0.05). In the marble burying test, SRF KO animals exhibited deficits, in which they buried fewer marbles than WT littermate controls (Fig. 5d, e; Mann–Whitney test, *p* < 0.01). Finally, SRF KO mice exhibited impairments in nest building compared with WT littermates (Fig. 5f, g; Mann–Whitney test, *p* < 0.01).

## Discussion

The present study assessed morphological, electrophysiological, and behavioral effects of SRF deficiency in adult excitatory granular neurons. The depletion of SRF in adult neurons leds to the direct downregulation of mRNAs of two actin-encoding genes and the functional inhibition of cofilin 1. Although alterations of actins and actin-binding protein did not alter gross hippocampal architecture, changes in dendritic spine morphology and basal synaptic transmission in granular neurons of the DG were observed. Moreover, SRF KO animals exhibited hyperactivity and impairments in behaviors that are typical for small rodents, recognized as hippocampus-dependent tasks. Altogether, our results suggest that basal SRF expression in adult DG neurons is engaged in the regulation of structural plasticity, basal synaptic transmission, and species-typical behaviors.

Alterations of spine morphology without gross changes in hippocampal architecture were the most prominent phenotype we identified in animals with a decrease in SRF expression in adult neurons. At the molecular level, we observed a decrease in the expression of neuronal actins and an increase in the phosphorylation of the actin-depolymerization factor cofilin 1. The actin cytoskeleton in neurons is composed of two distinct isoforms, non-muscle γ-actin and β-actin. Both actin genes are SRF targets and are downregulated in DG neurons in SRF KO animals (Miano et al. 2007; Sun et al. 2006). The present results confirmed earlier findings. The expression of both β-actin and γ-actin is impaired upon SRF ablation. We essentially generated the double knockdown of brain-expressed actin isoforms in *Srf* mouse mutants.

We also showed that the depletion of SRF in adult neurons coincided with an increase in cofilin 1 phosphorylation at Ser3 (inactivation), suggesting that in wild-type cells, SRF is involved in cofilin activation. Cofilin 1 activity could be inhibited by increased phosphorylation by LIM kinases (LIMKs) or decreased activity of phosphatases like slingshot (SSH) family of protein phosphatases (Mizuno 2013). In MRTFs-deficient neurons (myocardin-related transcription factors; SRF coactivators), a decrease in Pctaire1 kinase activity was shown to stimulate the Pak1-Limk cascade to promote cofilin phosphorylation (Mokalled et al. 2010). Pctaire1 (Cdk16) gene is a direct target of SRF/MRTF (Mokalled et al. 2010). Moreover, the decrease in actin levels observed in SRF-deficient neurons might impair slingshot activation, which is stimulated by association with actin filaments (Kurita et al. 2008; Liu et al. 2007; Nagata-Ohashi et al. 2004). Both mechanisms of increased cofilin 1 phosphorylation observed in SRF-deficient neurons are possible. Collectively, our data are consistent with a previous study that reported higher levels of phosphorylated cofilin 1 in SRF- or MRTFs -depleted neurons (Alberti et al. 2005; Beck et al. 2012; Mokalled et al. 2010; Zimprich et al. 2017).

In the present study, we observed a sharp reduction of total actin levels in SRF-depleted neurons. Interestingly, SRF deletion in non-neuronal cells resulted in the downregulation of β-actin and a decrease in the ratio of polymerized F-actin to G-actin (Randrianarison-Huetz et al. 2018; Schratt et al. 2002; Taylor et al. 2014). Thus, changes in spine morphology in SRF-depleted neurons could be attributable to both lower total actin expression and alterations of the regulation of actin polymerization. However, further quantification of the F-actin-to-G-actin ratio in neurons is needed. The link between SRF and actin is multifaceted. Actin is a direct target of SRF and controls SRF-dependent gene expression (Sotiropoulos et al. 1999). The conversion of monomeric G-actin to polymeric F-actin results in the activation of SRF-dependent transcription that is regulated by the translocation of MRTFs (Kalita et al. 2006; Kaneda et al. 2018) from the cytoplasm to the nucleus (Miralles et al. 2003; Posern et al. 2002, 2004). Moreover, nuclear actin dynamics were also shown to regulate the activity of SRF/MRTFs (Baarlink et al. 2013; Ho et al. 2013; Lundquist et al. 2014; Vartiainen et al. 2007). Thus, there is a feedback loop between SRF and actin, in which the activity of SRF regulates actin levels, the dynamics of which influence the transcriptional activity of SRF.

Actin cytoskeleton remodeling, stabilization, or depolymerization is crucial for dendritic spine morphology and synaptic function (Ethell and Pasquale 2005; Honkura et al. 2008; Matus 2000). Modifications of the number of neuronal connections and the size and shape of dendritic spines underlie synaptic plasticity (Borczyk et al. 2019; Holtmaat and Svoboda 2009; Kasai et al. 2010). Alterations of spine morphology underlie many neuropsychiatric disorders, indicating their functional importance (Glausier and Lewis 2013; Penzes et al. 2011; Phillips and Pozzo-Miller 2015). Spine parameters, such as the dimension of the spine head or neck, determine spine motility and stability (Nimchinsky et al. 2002). Mushroom-shaped spines are generally considered mature and stable. In contrast, thin spines with a long neck are highly plastic (Bourne and Harris 2007). In the present study, we found that SRF depletion in granular neurons shifted the spine population toward long spines with a less mature phenotype. Thus, dendritic spines grew but did not form enlarged heads, which are characteristic of mature mushroom spines. In addition to spine morphology, we observed deficits in basal synaptic transmission in SRF-depleted neurons. The lower amplitude (mostly mEPSCs of small and medium amplitudes) and frequency of mEPSCs that were observed in DG neurons that lacked SRF could represent either a change in release probability or postsynaptic processes, such as the loss of functional synapses.

Using several behavioral tasks, we showed that animals that lacked SRF protein in adult forebrain neurons built poorer nests, performed fewer digging bouts, exhibited a reduction of burying behavior compared with their WT littermates. In agreement with other studies, mice lacking SRF were significantly more active than control animals in the open field test (Johnson et al. 2011; Parkitna et al. 2010; Zimprich et al. 2017). Similar alterations in innate behaviors and activity were observed in animals with hippocampal lesions (Deacon et al. 2002). Deficits in nest building, digging and marble burying, in SRF KO animals, were also present in the animal models with smaller volume of the DG and abnormal spine morphology of DG neurons (Kondratiuk et al. 2013, 2017) and in mice with deficits in DGs adult neurogenesis (Jedynak et al. 2012). Although behaviors observed in SRF KO animals may reflect dysfunction of the dentate gyrus, we cannot exclude the possibility that SRF deletion in other brain structures impacts the observed phenotype. Interestingly, marble burying and nest building tests are interpreted by some authors as a measure of repetitive or social behaviors, respectively, were found to be altered in several murine models of psychiatric disorders, such as schizophrenia and autism spectrum disorders (Crawley 2012; Deacon 2006; Deacon et al. 2002; Jirkof 2014).

Loss-of-function of the *ACTB* gene in humans is associated with developmental delays or intellectual disability (Cuvertino et al. 2017; Palumbo et al. 2017). Moreover, a single-nucleotide polymorphism (SNP) of the *ACTB* gene that caused missense mutations was shown to be linked with several neurological phenotypes, including global developmental delay, intellectual disability, cognitive impairment, abnormal aggressive and impulsive behavior, attention deficit, hyperactivity, and autism spectrum disorders (based on DECIPHER database; http://www.decipher.sanger.ac.uk/, accessed 2018). An increase in hyperactivity and deficits in species-typical behaviors in SRF KO animals also resemble the phenotype of mice with the brain-specific deletion of β-actin, potentially linking some of the observed phenotypes in SRF-deficient neurons to the specific deletion of β-actin (Cheever et al. 2012). However, further work is needed to determine whether the specific depletion of β-actin and γ-actin in adult neurons mimics the phenotype that we observed in SRF KO animals. Overall, the present data support the notion that such behaviors as nest building, marble burying, and locomotor activity require intact hippocampal circuity.

Several lines of evidence suggest a role for SRF in brain pathology, such as epileptogenesis, cocaine-induced gene expression, and cocaine-induced dendritic spine formation (Cahill et al. 2017; Kuzniewska et al. 2016; Losing et al. 2017; Parkitna et al. 2010). Data also link SNPs of the *Srf* and *Mrtfs* genes to brain diseases. In humans, SNPs in CArG box (SRF transcription factor binding site), that disrupt SRF binding, were linked with neurological disorders, such as bipolar disorder, amyotrophic lateral sclerosis, and Alzheimer’s disease (Benson et al. 2011). Moreover, SNPs of the *Mrtfs* genes were associated with neurodevelopmental disorders, such as schizophrenia and autism spectrum disorders (Holt et al. 2010; Luo et al. 2015; Wang et al. 2016).

Altogether, our data indicate that the adult deletion of SRF in DG neurons alters the expression of actins, dendritic spine morphology, and mouse behavior, showing that SRF expression is necessary to maintain proper brain circuitry. To our knowledge, this is the first report that demonstrates that SRF regulates the adult structural plasticity of intact, adult hippocampal neurons in vivo. Furthermore, the present results link aberrant spine morphology to changes in glutamatergic synaptic transmission and behavior.
